# Perimenopausal syndrome and mood disorders in perimenopause: prevalence, severity, relationships, and risk factors

**DOI:** 10.1097/MD.0000000000004466

**Published:** 2016-08-12

**Authors:** Rui-xia Li, Min Ma, Xi-rong Xiao, Yan Xu, Xiu-ying Chen, Bin Li

**Affiliations:** a Department of Obstetrics and Gynecology, Obstetrics and Gynecology Hospital of Fudan University; b Shanghai Key Laboratory of Female Reproductive Endocrine Related Diseases, Shanghai, China.

**Keywords:** anxiety, depression, epidemiology, mood disorders, perimenopausal syndrome, perimenopause, risk factors

## Abstract

Limited information was focused on perimenopausal syndrome and mood disorders (depression and anxiety) in a specific population: perimenopausal women. We aimed to investigate the prevalence and severity of perimenopausal syndrome and mood disorders, and to analyze their relationships and risk factors in perimenopausal women in Shanghai, China.

A cross-sectional study was performed on 1062 women aged 40 to 60 years from 3 communities. The general conditions questionnaire, Kupperman index, self-rating depression scale, and self-rating anxiety scale were used. A multivariable logistic regression analysis was performed to identify risk factors for perimenopausal syndrome and mood disorders.

The prevalence of perimenopausal syndrome, depression and anxiety, which were primarily associated with mild symptoms, was 10.92%, 25.99%, and 12.62%, respectively. The differences in the prevalence and severity of perimenopausal syndrome, in the prevalence of depression, and in the severity of anxiety in different age groups were statistically significant (*P* *<* 0.001, *P* *=* 0.028, *P* *=* 0.003, *P* *=* 0.002, respectively). The relationships between perimenopausal syndrome and mood disorders were strong and positive (*P* *<* 0.001*)*. It was found that age, employment status, personality characteristics, menstruation, and constipation were risk factors for perimenopausal syndrome, but monthly household income was a protective factor. Also, higher income and better medical insurance were beneficial to depression. However, disharmonious family relationships, irregular menstruation, constipation, and severity of perimenopausal syndrome were harmful to depression. For anxiety, attitudes to children status, cesarean section times, and constipation were risk factors.

We concluded that perimenopausal syndrome and mood disorders are common in perimenopausal women in Shanghai, whose associations are strong and positive. Many risk factors are associated with and shared between perimenopausal syndrome and mood disorders. Therefore, appropriate management of perimenopause is needed to alleviate the conditions.

## 1 Introduction

Our population is aging and the proportion of people above 60 years of age is increasing and is accompanied by declining fertility rates.[ ^1^ ^2^] This is particularly the case for women, as their life expectancy is 6 to 8 years longer than that of men. ^[1]^ However, women tend to live longer and suffer from more diseases and disabilities, which have been related to a key transitional period that women experience in midlife: menopause.

Currently, the concept of “menopause” has been replaced by a more accurate term: “perimenopause.” Perimenopause involves three stages: premenopause (regular menstrual cycles with ≥12 menstruations during the past 12 months), menopausal transition (several menstruations but <12 during the past 12 months), and early postmenopause (no menstruations during the past 12 months). [^3^ ^4^ ^5^] Perimenopause is a natural physiological event that occurs in women and is defined by the World Health Organization (WHO) as the permanent cessation of menstruation and a decrease in the levels of ovarian steroid hormones (estrogen and progesterone) due to the loss of ovarian follicular function. The final menstrual period is retrospectively assigned after 12 consecutive months of amenorrhea in the absence of other pathological or physiological causes.[ ^2^ ^6^] It generally occurs around the age of 50 years, with a range between 40 and 60 years worldwide. [^6^ ^7^ ^8^ ^9^ ^10^ ^11^ ^12^] During perimenopause, women might experience symptoms such as hot flashes and night sweats, insomnia, vaginal dryness, mood disorders, and so on.[ ^8^ ^12^ ^13^ ^14^] Although most symptoms are not life-threatening, they may actually have a negative impact on the quality of life and the physical and mental health of perimenopausal women. ^[15]^ However, few studies have focused on the epidemiology of perimenopausal syndrome.

Depression and anxiety are common mood disorders. Depression, affecting approximately 350 million people, is the most common illness worldwide; in addition, the burden of depression has continued to rise globally. ^[16]^ The association between perimenopausal syndrome and depression has been studied extensively, but inconsistent results have been reported. [^17^ ^18^ ^19^] Therefore, an intrinsic link between depression and perimenopausal syndrome needs clarification. In addition, with the exception of the study by Terauchi et al, ^[20]^ few studies have focused on the relationship between anxiety and perimenopausal syndrome. They are precisely what we want to elucidate.

In this study, we aimed to investigate the prevalence and severity of perimenopausal syndrome and mood disorders, analyze the relationships between perimenopausal syndrome and mood disorders, and reveal risk factors for perimenopausal syndrome and mood disorders.

## 2 Materials and methods

### 2.1 Inclusion and exclusion criteria for the study participants

The inclusion criteria included the following: women who met the standards of the stages of reproductive aging workshop (STRAW); women with a healthy body; and women with a healthy uterus and at least one healthy ovary. [^3^ ^4^ ^5^ ^6^] The exclusion criteria consisted of the following four parts: chronic irregular menstruation with pathological or physiological causes, hysterectomy, abnormal anatomical structure of the uterus or ovaries; treatment for severe psychiatric illness; hormone therapy within 3 months; and presence of diseases of the endocrine system like hyperthyroidism and others.

### 2.2 Criteria for body mass index

Body mass index (BMI) was divided into 4 grades according to the China's Ministry of Health Disease Control Department criteria, as follows: BMI < 18.5 (underweight), 18.5 ≤ BMI < 24 (normal), 24 ≤ BMI < 28 (overweight), and BMI ≥ 28 (obese). ^[5]^ The unit of BMI is kg/m^2^. These criteria were very consistent with the reference standards of WHO.

### 2.3 Study participants

The current study was approved by the Ethics Committee of Obstetrics and Gynecology Hospital of Fudan University. Informed consent was obtained from each enrolled participant.

**Figure 1:**
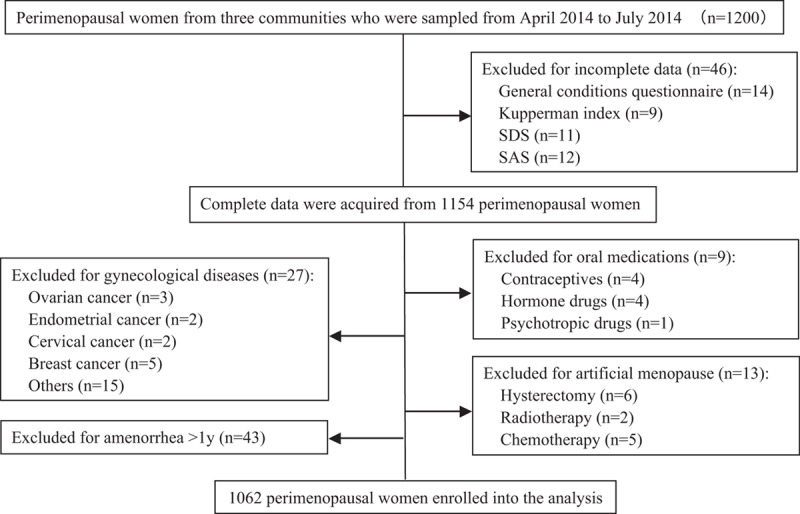
Flow chart for the selection of the study participants.

According to previous studies, a total of 1200 women aged 40 to 60 years from 3 communities in Shanghai were chosen between April 2014 and July 2014 using cluster sampling. Of these participants, 46 women whose questionnaires were incomplete (14 for general conditions questionnaire, 9 for Kupperman index, 11 for self-rating depression syndrome (SDS), and 12 for self-rating anxiety syndrome (SAS)), were excluded. In addition, we excluded those who were treated with oral medications (4 for contraceptives, 4 for hormone drugs, and 1 for psychotropic drugs) as well as those with gynecological diseases (3 for ovarian cancer, 2 for endometrial cancer, 2 for cervical cancer, 5 for breast cancer, and 15 for others), artificial menopause (6 for hysterectomy, 2 for radiotherapy, and 5 for chemotherapy), and amenorrhea more than 1 year (n = 43). Finally, 1062 perimenopausal women were enrolled in this analysis. A flow chart that depicts the selection of the study participants is shown in Fig. 1.

### 2.4 Procedures

A self-administered questionnaire that composed of 4 parts was generated after several discussions, including the general conditions questionnaire, the Kupperman index, SDS, and SAS. A pilot survey was conducted to test the questionnaire among 30 volunteers (patients, nurses, and doctors) at our clinic. The questionnaire was further refined before it was used in the actual study. All researchers underwent a training course and familiarized themselves with the questionnaire contents. Advocacy work toward the residents was performed by community staff, ensuring that this study was conducted on a voluntary basis. Explanations were given face-to-face when participants were confused of any questions.

### 2.5 Questionnaires

The general conditions questionnaire comprised up to approximately 20 items, which were age, body weight, height, education, monthly household income, employment status, medical insurance, marital status, menstrual history, reproductive history, personality characteristics, family relationships, smoking habits, and alcohol consumption, among others. This questionnaire was used to describe the sociodemographic and general characteristics of the study participants.

The Kupperman index has been widely used to evaluate perimenopausal syndrome, and this portion of the survey contained 11 items such as hot flashes and night sweats, paresthesias, insomnia, anxiety, depression, dizziness, fatigue, arthralgia and myalgia, headache, palpitations, and skin formication. ^[21]^ They were divided into 4 grades (0–3 points) according to the severity of the symptoms, as follows: 0, no; 1, mild; 2, moderate; 3, severe. The weighted scores for hot flashes and night sweats were 4 points; paresthesia, insomnia and anxiety were 2 points each; other symptoms were 1 point each. Each item score was calculated as the product of the weighted score multiplied by the points according to the severity. The total Kupperman score is the sum of the scores of all the items, and the highest possible score was 51 points. Perimenopausal syndrome was considered to be mild with a total Kupperman score of 15 to 20 points, moderate with 21 to 35 points, and severe with 36 to 51 points.

SDS, which included 20 items, was chosen to assess the severity of depression. ^[22]^ Each item was assigned to 4 grades (1–4 points) as follows: 1, none or seldom; 2, sometimes; 3, often; and 4, always. Ten items were statements of positive words for the reverse order scoring (4–1), and the remaining were negative statements with a sequence order scoring (1–4). We calculated the total SDS score as the sum of each item score. However, the standard total SDS score was the integer part of the total SDS score of 1.25 times. If the standard total SDS score was less than 50 points, the person was considered normal with respect to depression. Similarly, the depressive symptoms were considered mild with a standard total score of 50 to 59 points, moderate with 60 to 69 points, and severe with 70 points or more.

The symptoms of anxiety were evaluated by SAS. ^[23]^ This questionnaire also consisted of 20 items, as follows: 15 negative statements with a sequence order scoring (1–4) and 5 positive statements with a reverse order scoring (4–1). The calculation method for the standard total SAS score and the evaluation criteria for the severity of anxiety were the same as those for SDS. The cut-points in the manuscript for Kupperman index, SDS, and SAS were determined according to previous references. [^21^ ^22^ ^23^]

### 2.6 Data collection

Two researchers (R-xL and MM) strictly screened all questionnaires and excluded the questionnaires with incomplete data. Data were extracted twice by Epidata 3.1 software (Epidata association, Denmark) to ensure accurate data.

### 2.7 Quality control

The questionnaires were designed by extensive referrals to the literature and were further refined after a presurvey and repeated discussions. A 6-hour training course was provided to all researchers so that they were all familiar with the contents of the questionnaires. Study participants were randomly selected and this study was conducted on a voluntary basis.

### 2.8 Statistical analysis

The SPSS version 16.0 statistical software package (SPSS Inc., Chicago, IL) was used for data analysis. Descriptive statistics were used to summarize the characteristics of the participants, the prevalence and severity of perimenopausal syndrome and mood disorders, and the frequency of each perimenopausal symptom and system. Continuous data were expressed as mean (SD). Comparisons of continuous variable between 2 groups were performed by *t*-test for equal variances or Wilcoxon rank sum test for unequal variances. A one-way analysis of variance (ANOVA) and the Kruskal–Wallis rank sum test were used for comparisons of 3 or more study groups. The χ^2^ test and Fisher exact test were used to compare categorical variables. The relationships between perimenopausal syndrome and mood disorders were assessed by χ^2^ test. The risk factors for perimenopausal syndrome and mood disorders were identified by multivariable logistic regression analysis. The results are presented as an odds ratio (OR) with 95% confidence interval (CI). *P* < 0.05 was defined as statistically significant.

## 3 Results

### 3.1 General characteristics of the participants

The general characteristics of the participants are demonstrated in detail in Table 1 . The mean age was 49.66 ± 4.24 years, and most (78.72%) were 45 to 55 years old. The mean BMI was 22.90 ± 2.53 kg/m^2^, and 67.98% of them were considered as normal (21.73 ± 1.41 kg/m^2^). Most participants were employed or retired (91.15%), had medical insurance (86.06%), were married and cohabiting (94.73%), and had 2 to 4 family members (83.99%). A harmonious family relationship accounted for 84.93%, and 98.87% of this population were very satisfied or satisfied with their childbearing status. Most participants demonstrated characteristics of an outgoing personality (76.27%), regular menstruation (53.11%), a history of 1 to 2 pregnancies (91.53%), and 1 to 2 vaginal deliveries (78.15%). However, few of the participants (14.12%) were highly educated (at least a college education or above), and the percentage of participants in China with an average monthly household income over 5000 yuan was only 26.55%. In addition, most of the study population had never had an abortion (68.17%), a cesarean section (78.91%), constipation (78.91%), had no history of smoking (99.06%) or alcohol consumption (89.83%). The participants were divided into 2 groups according to the total Kupperman score: 116 (10.92%) had perimenopausal syndrome (perimenopausal syndrome group) and 946 (89.08%) did not have perimenopausal syndrome (non-perimenopausal syndrome group). Some items, such as the age at marriage, BMI, education, medical insurance, marital status, number of family members, reproductive history, the birth weight of newborns, and smoking habits, were similar between the 2 groups.

**Table 1:**
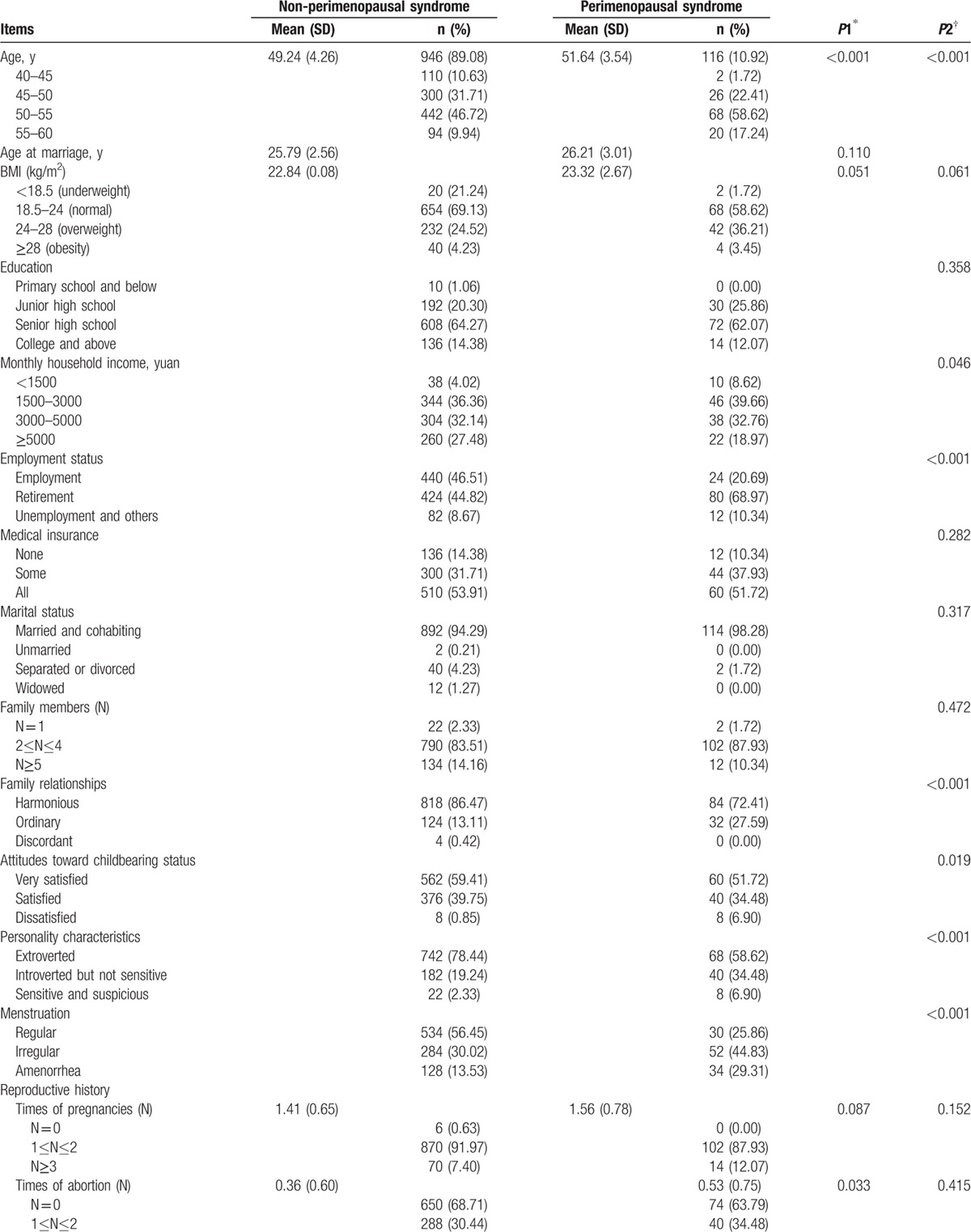
General characteristics of perimenopausal women (n = 1062).

**Table 1 (Continued):**
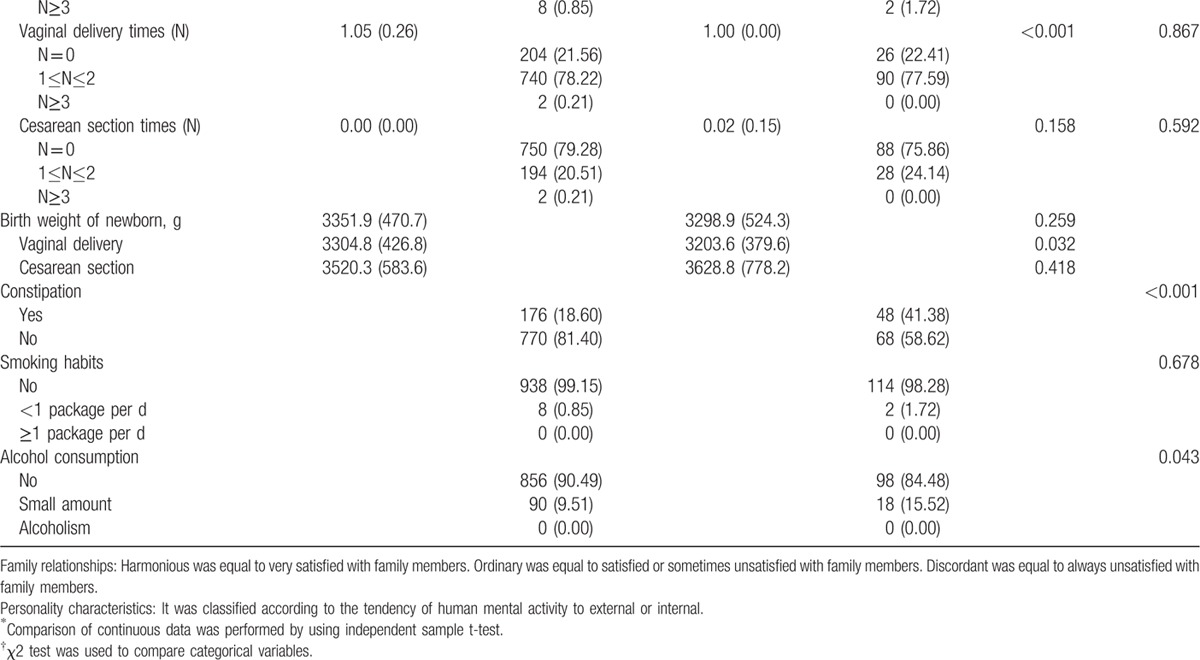
General characteristics of perimenopausal women (n = 1062).

### 3.2 Prevalence and severity of perimenopausal syndrome

The prevalence and severity of perimenopausal syndrome in the different age groups are shown in Table 2. It was found that 10.92% of respondents had perimenopausal syndrome. In terms of total morbidity, most participants with perimenopausal syndrome had mild (75.86%) or moderate symptoms (24.14%), while no severe symptoms were found in any respondents to this survey. The prevalence of perimenopausal syndrome in the different age groups (1.79%, 7.98%, 13.33%, and 17.54% in the 40–45, 45–50, 50–55, and 55–60 age groups, respectively) demonstrated a statistically significant difference (*P* *<* 0.001), which suggests that the frequency of perimenopausal syndrome increases with advancing age. The results of multiple comparisons of the prevalence of perimenopausal syndrome in the different age groups showed significant differences (50–55 age group vs 40–45 age group, *P* *<* 0.001; 55–60 age group vs 40–45 age group, *P* *<* 0.001; 55–60 age group vs 45–50 age group, *P* *=* 0.004). We also found a statistical significance among the severity of perimenopausal syndrome in the different age groups (*P* *=* 0.028), and multiple comparisons showed a significant difference (55–60 age group vs 40–45 age group, *P* *=* 0.002). Furthermore, in terms of the severity of perimenopausal syndrome, those in all age groups had primarily mild, non-severe symptoms.

**Table 2:**
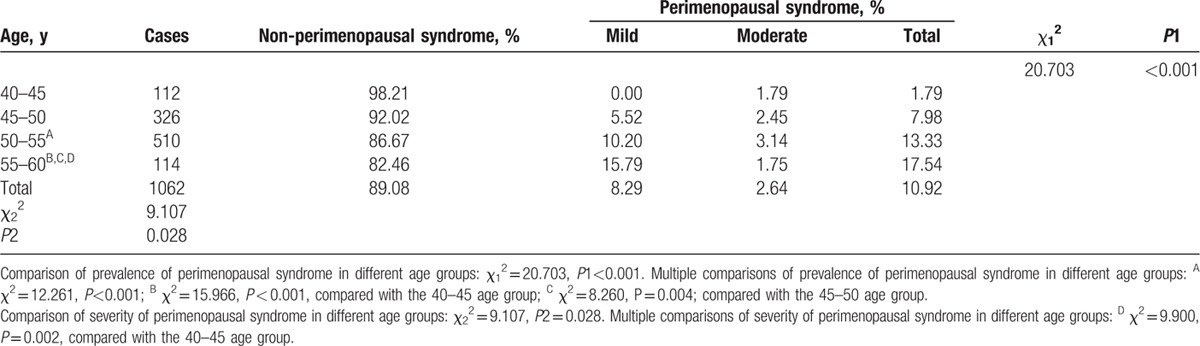
Prevalence and severity of perimenopausal syndrome in different age groups.

### 3.3 Perimenopausal symptoms

Seventy-six percent or more of the participants experienced at least one perimenopausal symptom. Table 3 depicts the proportion of each perimenopausal symptom in descending order. The most common symptom experienced by perimenopausal women was fatigue (54.24%), which was the only single symptom that accounted for more than 50% of all symptoms. The order of the symptoms according to their proportion was dizziness (44.63%), insomnia (40.68%), headache (38.98%), arthralgia and myalgia (37.48%), hot flashes and night sweats (32.02%), palpitations (31.45%), anxiety (24.11%), paresthesias (22.60%), depression (13.18%), and skin formication (8.85%). Hot flashes and night sweats as well as anxiety and depression were separately ranked sixth, eighth and tenth. The rarest symptom was skin formication.

**Table 3:**
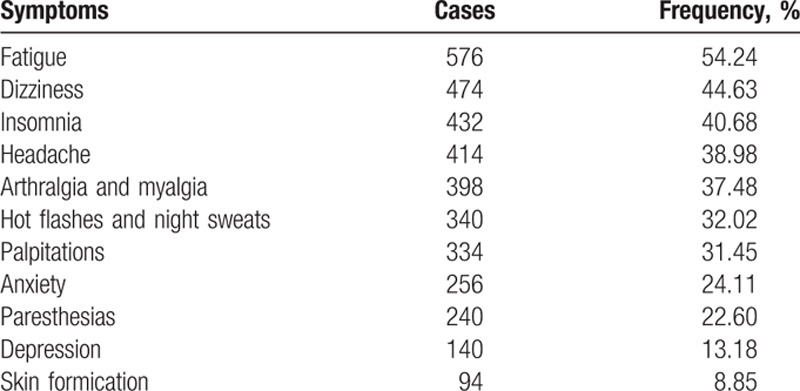
Frequency of perimenopausal symptoms of perimenopausal women (n = 1062).

### 3.4 Different systems of perimenopausal symptoms

Table 4 describes the prevalence of different systems of perimenopausal symptoms in perimenopausal women of different age groups. The perimenopausal symptoms were assigned to the following 4 categories: vasomotor system (e.g., hot flashes and night sweats), neuropsychiatric system (e.g., paresthesias, insomnia, anxiety, depression, and skin formication), cardiovascular system (e.g., dizziness, headache, and palpitations), and skeletal system (e.g., fatigue, arthralgia, and myalgia). The sequence of each system according to descending prevalence was skeletal system (68.83%), neuropsychiatric system (52.17%), cardiovascular system (50.19%), and vasomotor system (32.03%). The prevalence of each system in different age groups was significantly different (*P* *<* 0.001, *P* *=* 0.036, *P* *<* 0.001, and *P* *<* 0.001, respectively). For vasomotor and skeletal system, the results of multiple comparisons showed a statistically significant difference compared with the 40 to 45 age group (45–50 age group, *P* *=* 0.009 and *P* *<* 0.001; 50–55 age group, *P* *<* 0.001 and *P* *<* 0.001; 55–60 age group, *P* *<* 0.001 and *P* *<* 0.001, respectively) and the 45 to 50 age group (50–55 age group, *P* *<* 0.001 and *P* *=* 0.001; 55 to 60 age group, *P* *<* 0.001 and *P* *=* 0.012, respectively). In regards to cardiovascular system, the proportion in the 45 to 50 and 55 to 60 age groups was significantly higher than that of the 50 to 55 age group (*P* *<* 0.001). However, the proportion was similar among different age groups with respect to neuropsychiatric system. With the exception of cardiovascular system, the prevalence of other systems with an increasing trend was observed with advancing age.

**Table 4:**
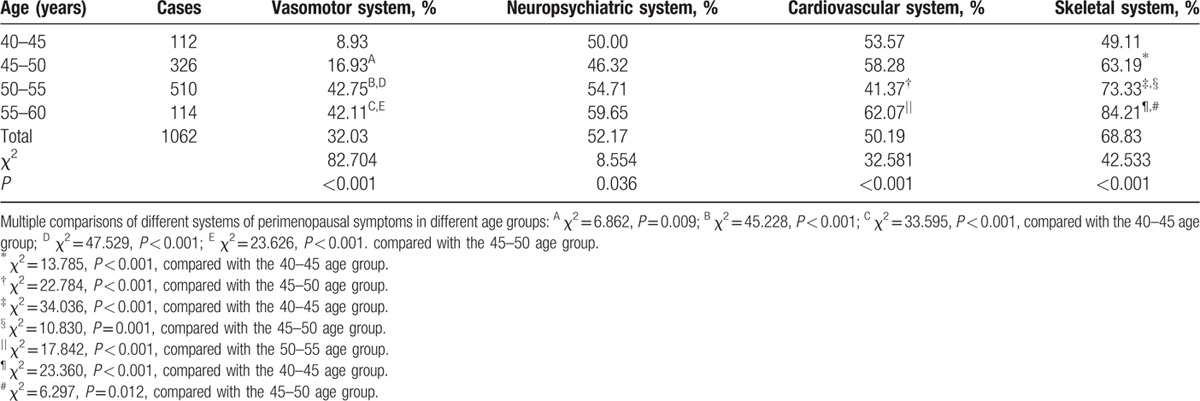
Prevalence of different systems of perimenopausal symptoms of perimenopausal women in different age groups.

### 3.5 Prevalence and severity of mood disorders

The results of the prevalence and severity of perimenopausal depression according to SDS in the different age groups are listed in Table 5. Overall, 25.99% of participants experienced depression. Most women had mild (16.01%) or moderate (9.79%) depression, whereas only 0.19% experienced severe symptoms. Obviously, the difference in the prevalence of depression among the 4 age groups was significant (*P* *=* 0.003). The prevalence was lowest in the 55 to 60 age group, which was significantly decreased compared with the prevalence in the 50 to 55 age group (*P* *<* 0.001). However, the severity of depression among the 4 age groups was similar (*P* *=* 0.331).

**Table 5:**
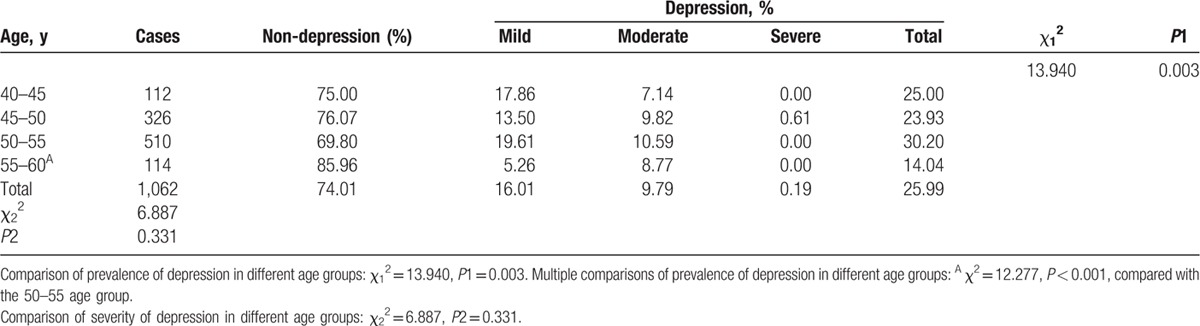
Prevalence and severity of perimenopausal depression by self-rating depression syndrome (SDS) in different age groups.

Table 6 summarizes the prevalence and severity of perimenopausal anxiety by SAS in the different age groups. According to the survey, the prevalence of anxiety was 12.62%. Among these participants, 11.11% experienced mild symptoms, 1.32% experienced moderate symptoms, and 0.19% experienced severe symptoms. The prevalence of anxiety in each age group was no different (*P* *=* 0.169). However, the severity of the anxiety among the 4 age groups was significantly different (*P* *=* 0.002). Multiple comparisons of the severity of anxiety in the different age groups showed that the difference was statistically significant (50–55 age group vs 40–45 age group, *P* *=* 0.007; 55–60 age group vs 50–55 age group, *P* *=* 0.002).

**Table 6:**
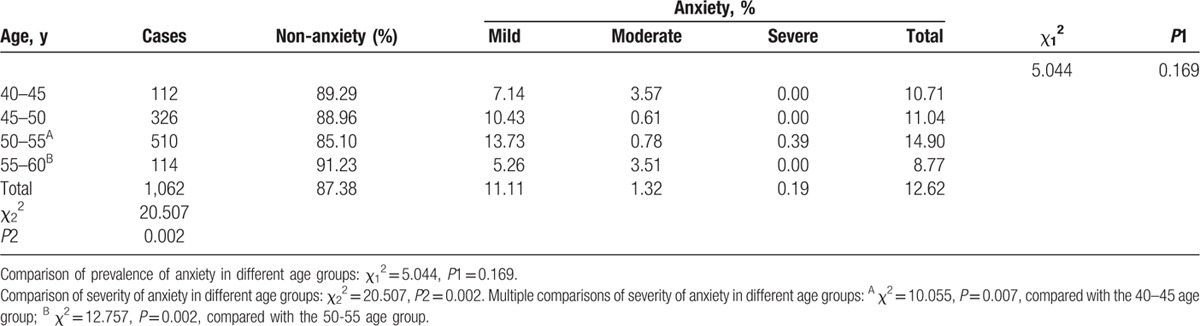
Prevalence and severity of perimenopausal anxiety by self-rating anxiety syndrome (SAS) in different age groups.

### 3.6 Relationships between perimenopausal syndrome and mood disorders

Table 7 shows the relationships between perimenopausal syndrome and mood disorders. The prevalence of depression in the perimenopausal syndrome group was significantly higher than that in the non-perimenopausal syndrome group (51.72% vs 22.83%, *P* *<* 0.001). Additionally, a positive correlation was observed between perimenopausal syndrome and depression (*P* *<* 0.01). Most likely, comparisons of the severity of depression in each perimenopausal syndrome subgroup were significantly different (*P* < 0.001). Therefore, it was suggested that the severity of depression in perimenopausal women was related to the severity of perimenopausal syndrome, and a positive correlation was found (*P* *<* 0.01).

**Table 7:**
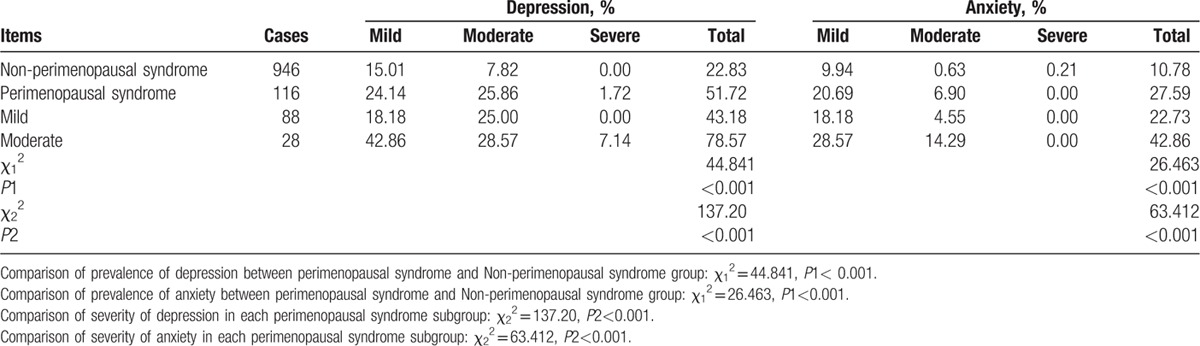
The relationships between perimenopausal syndrome and mood disorders.

In regards to anxiety, 27.59% of the participants in the perimenopausal syndrome group experienced this mood disorder whereas only 10.78% of the participants in the non-perimenopausal syndrome group experienced this disorder. However, a significant difference (*P* *<* 0.001) and a positive correlation *(P* *<* 0.01) were found between perimenopausal syndrome and anxiety. Furthermore, the results of comparisons of the severity of anxiety in each perimenopausal syndrome subgroup were consistent with those of depression, which indicates that the difference was significant (*P* *<* 0.001) and that the correlation was positive (*P* *<* 0.01).

### 3.7 Risk factors for perimenopausal syndrome and mood disorders

In regards to the univariable analysis, our survey revealed that age, monthly household income, employment status, family relationships, attitudes toward childbearing status, personality characteristics, menstruation, times of abortion, vaginal delivery times, constipation, and alcohol consumption were significantly associated with perimenopausal syndrome. However, BMI, education level, and smoking habits, among other factors, had no statistically significant association with perimenopausal syndrome. All of these factors are shown in Table 1 . In regards to depression, it was found that monthly household income, medical insurance, family relationships, menstruation, constipation, perimenopausal syndrome, and severity of perimenopausal syndrome demonstrated a statistically significant association. Additionally, we found attitudes toward childbearing status, cesarean section times, perimenopausal syndrome, and severity of perimenopausal syndrome had a significant association with anxiety. However, after a multivariable analysis—stepwise regression, the risk factors in Tables 8–10 were clearly demonstrated and the results were all adjusted. Age, employment status, personality characteristics, menstruation, and constipation were risk factors for perimenopausal syndrome, but monthly household income was a protective factor. Additionally, we found that higher income and better medical insurance were beneficial to depression. In contrast, disharmonious family relationships, irregular menstruation, constipation, and the severity of perimenopausal syndrome were risk factors for depression. In terms of anxiety, attitudes toward childbearing status, cesarean section times, and constipation were risk factors.

**Table 8:**
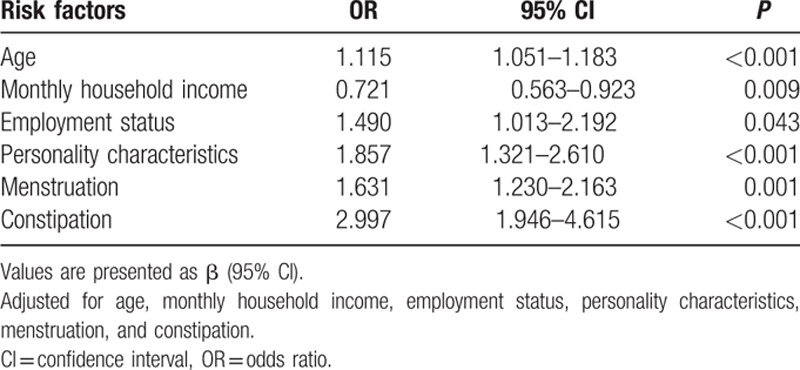
The results of multivariable logistic regression analysis of risk factors of perimenopausal syndrome.

**Table 9:**
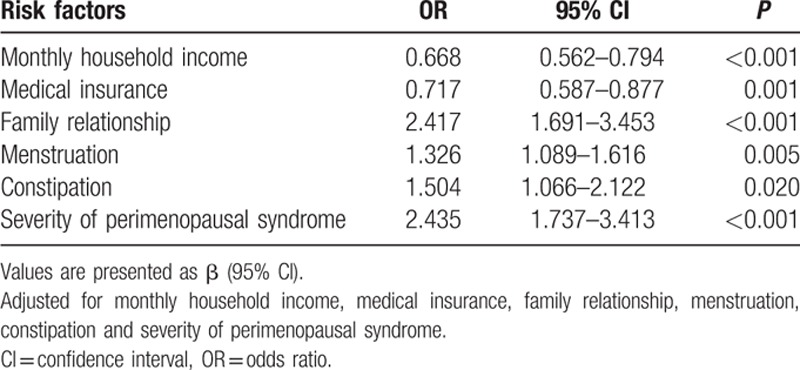
The results of multivariable logistic regression analysis of risk factors of depression.

**Table 10:**
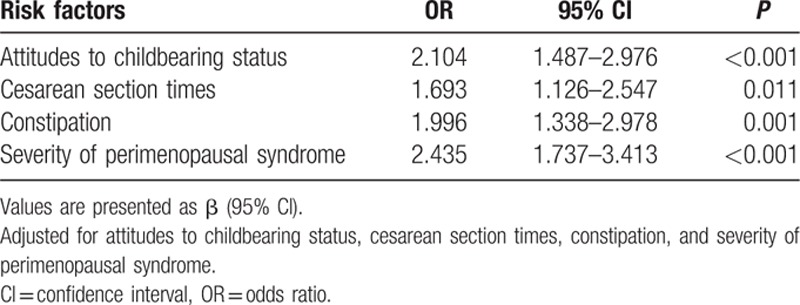
The results of multivariable logistic regression analysis of risk factors of anxiety.

## 4 Discussion

The main purpose of this article was to describe the prevalence and severity of perimenopausal syndrome and mood disorders in Shanghai, China, to elucidate the relationships between them and to reveal risk factors for both of these conditions. We found that the prevalence of perimenopausal syndrome, depression, and anxiety, which were all primarily associated with mild symptoms, was 10.92%, 25.99%, and 12.62%, respectively. The differences in the prevalence and severity of perimenopausal syndrome, in the prevalence of depression, and in the severity of anxiety in different age groups were statistically significant. The relationships between perimenopausal syndrome and mood disorders were strong and positive. Many common factors were associated with and shared between perimenopausal syndrome and mood disorders.

The sociodemographic characteristics (e.g., education, income, and race/ethnicity) were different in developing and developed countries.[ ^8^ ^9^ ^17^ ^18^ ^24^] The average age of our participants was close to 50 years, which was in accordance with most studies in Latin America and in Asian countries such as China, Singapore, and Korea; however, the average age was slightly lower compared with most studies from the USA and Europe and was higher than studies that were conducted in Turkey and India.[ ^5^ ^8^ ^9^ ^17^ ^18^ ^24^ ^25^ ^26^ ^27^ ^28^ ^29^] This might not be explained by sociocultural differences or simply by genetic differences.

With advancing age, the prevalence of perimenopausal syndrome, especially perimenopausal syndrome with mild symptoms, increased gradually due to hormone deficiency. However, no women experienced severe perimenopausal syndrome in the present survey, which might be related to the sampling methodology, and thus further studies are needed to clarify this issue. We found that few studies had paid attention to perimenopausal syndrome, and instead, they cast some light on perimenopausal symptoms. Fortunately, a health survey, including 2100 registered nurses aged 40 to 55 from 20 hospitals in Beijing, showed that 37.83% of the participants who experienced perimenopause had perimenopausal syndrome. ^[5]^ This value was significantly higher than that in our study, which suggests that the work environment may play an important role in perimenopausal syndrome, but whether this is related to work pressure or the living environment requires further study.

Although the majority of women pass through perimenopause without difficulty, some women experience mood disorders during this time. According to SDS and SAS, 25.99% of the participants were diagnosed with depression while 12.62% were diagnosed with anxiety. Most of these cases were mild (16.01% for depression and 11.11% for anxiety). As is clearly shown, the prevalence of symptoms of depression was 13.18% and that of anxiety was 24.11% during perimenopause. A survey conducted in Guangdong province in southern China showed that 21.1% of women had experienced feelings of depression and 29.7% had experienced feelings of anxiety. ^[9]^ The prevalence of depressive symptoms among middle-aged women varies by country, with 24% in Beijing, China, 25% in Turkey, 31% to 38.7% in Taiwan, 40% in Spain, 50% in Philadelphia, USA, and 52% in Mexico, whereas few studies have focused on anxiety during perimenopause.[ ^14^ ^30^ ^31^ ^32^] The key problem was the disparity in the prevalence of mood disorders as determined by SDS, SAS, and the Kupperman index. The following 2 explanations may be useful: different questionnaires and different diagnostic criteria. Whether these explanations are valid or not, future studies are needed. Furthermore, the prevalence of mood disorders tended to decrease with increasing age, especially during postmenopause, which was similar to what was found in previous studies. ^[19]^

The association between perimenopausal syndrome and depression has been studied extensively, with inconsistent results. [^17^ ^18^ ^19^] In our current study, a positive association between perimenopausal syndrome and depression was found. A national prospective cohort study in Taiwan showed that symptomatic menopausal transition increased the risk of new-onset depressive disorder later in life. ^[18]^ Another population-based study in Taiwan also found that the increase in depressive symptoms was significantly associated with menopausal status and with the majority of menopausal symptoms. ^[17]^ Longitudinal studies have demonstrated an association between the menopausal transition and an increase in depressive symptoms. ^[16]^ These findings are similar to those of our survey. Nevertheless, contrary to widely held beliefs, perimenopause is not associated with an increase in psychiatric illness. ^[19]^ At the same time, we found a strong correlation between perimenopausal syndrome and anxiety. Although few studies have focused on the relationship between perimenopausal syndrome and anxiety, the survey by Terauchi et al ^[20]^ indirectly suggested that the correlation between perimenopausal syndrome and anxiety is positive and strong.

Many factors were found to be associated with perimenopausal syndrome, depression, and anxiety, although they were different. Our present survey suggests that age, employment status, personality characteristics, menstruation, and constipation are risk factors for perimenopausal syndrome, but that monthly household income is a protective factor. As illustrated in the tables, the higher the age, the higher the prevalence of perimenopausal syndrome. Irregular menstruation, due to hormonal changes, may result in perimenopausal syndrome. Although the reasons are not clear, the loss of a job, introverted or sensitive personality characteristics, and constipation all have an influence on perimenopausal syndrome to some degree. However, monthly household income showed a positive association with perimenopausal syndrome, which implies that the higher the income, the less likely the prevalence of perimenopausal syndrome. Recent unhappy events and ethnicity were associated with an increased risk of perimenopausal symptoms, ^[24]^ which were not reported in our survey. Therefore, further studies are warranted because the reasons that these factors might contribute to perimenopausal syndrome remain unclear, and other factors such as dietary pattern have not yet been investigated. However, a single-blind, randomized, placebo-controlled trial conducted in early postmenopausal Chinese women showed that soy isoflavones could attenuate bone loss. ^[21]^

We also revealed that higher income and better medical insurance were beneficial to depression, whereas disharmonious family relationships, irregular menstruation, constipation, and severity of perimenopausal syndrome were associated factors for depression. Higher income and better medical insurance were helpful for stress relief and for improvement in the quality of life, which in turn may have helped to reduce the occurrence of depression. This suggests that the more severe the perimenopausal syndrome, the higher the susceptibility to depression.

In regards to anxiety, attitudes toward childbearing status, cesarean section times, and constipation were associated factors. If women were not satisfied with their childbearing situations, they would be more likely to be anxious. Of course, the higher the frequency of cesarean section, the higher the frequency of anxiety among the women. Constipation was a shared risk factor for perimenopausal syndrome and mood disorders.

Therefore, appropriate measures should be taken to reduce the prevalence of perimenopausal syndrome and mood disorders in daily life. Knowledge about perimenopause should also be imparted to women in the community so that they may have a proper understanding of perimenopause. For these risk factors, women should be proactive (e.g., the establishment of harmonious relationships, positive attitudes, development of good life habits, hormone replacement therapy) to prevent or reduce the occurrence of perimenopausal syndrome and mood disorders.

As is known that health is a state of complete physical, mental and social wellbeing, an individual or group must be able to identify and to realize aspirations, to satisfy needs, and to change or cope with the environment as well as appropriate political and financial circumstances.[ ^33^ ^34^] In fact, various ways including the design of the setting could promote and maintain health, which indicates supporting health is a multidimensional act. ^[34]^ The term “setting” has an increasing influence on health promotion, which could be focused on the living areas like geography and ecology, the social institutions like school, university, and occupation, or the regional multidimensional context such as the state or city. ^[34]^ In cities, our basic necessities and problematic social circumstance could have a great impact on ways of living. ^[34]^ Therefore, the design of the setting, healthy or unhealthy cities, problem to national health resources, and loss of traditional situation could make severe alterations to women in health. Furthermore, social inequalities, involving socioeconomic status, education, health care, and so on, also have a significant influence on health. [^34^ ^35^ ^36^ ^37^ ^38^ ^39^ ^40^] An investigation about the magnitude of inequalities in mortality and self-assessed health among 22 European countries suggested that variation across Europe in the magnitude of inequalities in health associated with socioeconomic status and these inequalities might be reduced by improving educational opportunities, income distribution, health-related behavior, or access to health care. ^[35]^ In England, Demakakos et al ^[36]^ pointed out wealth appeared to be more strongly associated with mortality than other socioeconomic position measures. Among the developed countries, it is not the richest societies that have the best health, but those that have the smallest income differences between the rich and poor. ^[37]^ For inequalities in disability-free life expectancies among older people, a cross-sectional data (n = 32,724) from the WHO Study on global AGEing and adult health (SAGE) in China, Ghana, India, Mexico, the Russian Federation, and South Africa during 2007 and 2010 showed that the disability prevalence ranged from 13% in China to 54% in India. ^[38]^ A comparison of 26 European countries in 2009 revealed educational differentials in disability varied markedly. ^[39]^ In summary, the design of the setting and social inequalities had an influence on health, which should be paid more attention.

The current study has some strengths. First, this study comprehensively measured the prevalence, severity, relationships, and risk factors of perimenopausal syndrome and mood disorders. Second, the prevalence, severity, relationships, and risk factors of perimenopausal syndrome and mood disorders were first described in the same population. Previous studies only investigated some of these in a survey format.[ ^5^ ^8^ ^9^ ^24^] Third, with the exception of the general conditions questionnaire, all questionnaires used were common international rating scales. Fourth, we strictly adhered to good quality control measures.

The present study also has some limitations. First, as this was a cross-sectional survey, we were unable to determine a cause and effect relationship from such associations. In addition, the participants were selected from 3 small communities, which might limit the external validity of the results. Moreover, the determination of the frequency of perimenopausal symptoms and mood disorders was limited by self-reported data, and therefore, recall bias should be considered. Furthermore, mood disorders were assessed by questionnaires rather than by a diagnosis obtained from a clinical psychiatrist. Last but not least, the sample size was small, and a larger sample size might be needed.

## Acknowledgments

We would like to show our great appreciation to Shanghai Charity Foundation for funds. We are grateful to Dr Chao Gu, Qing Cong, Ge-Ting Zhu, Xi Chen, Ting Guo, Zi-Yang Lu, Hao Yu, and Lu Guo from Obstetrics and Gynecology Hospital of Fudan University for their help in the investigation. We also thank Yuan He from Obstetrics and Gynecology Hospital of Fudan University for evaluating statistical information reported.

## References

[1] Wang H Dwyer-Lindgren L Lofgren KT Age-specific and sex-specific mortality in 187 countries, 1970–2010: a systematic analysis for the Global Burden of Disease Study 2010. *Lancet* 2012; 380:2071–2094. 23245603 10.1016/S0140-6736(12)61719-X

[2] Jaspers L Daan NM van Dijk GM Health in middle-aged and elderly women: a conceptual framework for healthy menopause. *Maturitas* 2015; 81:93–98. 25813865 10.1016/j.maturitas.2015.02.010

[3] Harlow SD Gass M Hall JE Executive summary of the stages of reproductive aging workshop + 10: addressing the unfinished agenda of staging reproductive aging. *J Clin Endocr Metab* 2012; 97:1159–1168. 22344196 10.1210/jc.2011-3362 PMC3319184

[4] Soules MR Sherman S Parrott E Executive summary: Stages of Reproductive Aging Workshop (STRAW). *Climacteric* 2001; 4:267–272. 11770182

[5] Liu M Wang Y Li X A health survey of Beijing middle-aged registered nurses during menopause. *Maturitas* 2013; 74:84–88. 23149163 10.1016/j.maturitas.2012.10.006

[6] Hulka BS Meirik O Research on the menopause. *Maturitas* 1996; 23:109–112. 8735349 10.1016/0378-5122(95)00967-1

[7] Treloar AE Boynton RE Behn BG Variation of the human menstrual cycle through reproductive life. *Int J Fertil* 1967; 12:77–126. 5419031

[8] Yim G Ahn Y Chang Y Prevalence and severity of menopause symptoms and associated factors across menopause status in Korean women. *Menopause* 2015; 22:1–9. 25783469 10.1097/GME.0000000000000438

[9] Yang D Haines CJ Pan P Menopausal symptoms in mid-life women in southern China. *Climacteric* 2008; 11:329–336. 18645699 10.1080/13697130802239075

[10] Waidyasekera H Wijewardena K Lindmark G Menopausal symptoms and quality of life during the menopausal transition in Sri Lankan women. *Menopause* 2009; 16:164–170. 18703984 10.1097/gme.0b013e31817a8abd

[11] Grigoriou V Augoulea A Armeni E Prevalence of vasomotor, psychological, psychosomatic and sexual symptoms in perimenopausal and recently postmenopausal Greek women: association with demographic, life-style and hormonal factors. *Gynecol Endocrinol* 2013; 29:125–128. 22849709 10.3109/09513590.2012.708801

[12] Moilanen J Aalto AM Hemminki E Prevalence of menopause symptoms and their association with lifestyle among Finnish middle-aged women. *Maturitas* 2010; 67:368–374. 20869181 10.1016/j.maturitas.2010.08.007

[13] El Shafie K Al Farsi Y Al Zadjali N Menopausal symptoms among healthy, middle-aged Omani women as assessed with the Menopause Rating Scale. *Menopause* 2011; 18:1113–1119. 21844827 10.1097/gme.0b013e31821b82ee

[14] Perez JA Garcia FC Palacios S Epidemiology of risk factors and symptoms associated with menopause in Spanish women. *Maturitas* 2009; 62:30–36. 19010615 10.1016/j.maturitas.2008.10.003

[15] Greenblum CA Rowe MA Neff DF Midlife women: symptoms associated with menopausal transition and early postmenopause and quality of life. *Menopause* 2013; 20:22–27. 22929034 10.1097/gme.0b013e31825a2a91

[16] Vivian-Taylor J Hickey M Menopause and depression: is there a link? *Maturitas* 2014; 79:142–146. 24951102 10.1016/j.maturitas.2014.05.014

[17] Lin H Hsiao M Liu Y Perimenopause and incidence of depression in midlife women: a population-based study in Taiwan. *Climacteric* 2013; 16:381–386. 22963154 10.3109/13697137.2012.707706

[18] Chen MH Su TP Li CT Symptomatic menopausal transition increases the risk of new-onset depressive disorder in later life: a nationwide prospective cohort study in Taiwan. *PLoS One* 2013; 8:e59899. 23544108 10.1371/journal.pone.0059899 PMC3609738

[19] Robinson GE Psychotic and mood disorders associated with the perimenopausal period: epidemiology, aetiology and management. *CNS Drugs* 2001; 15:175–184. 11463126 10.2165/00023210-200115030-00002

[20] Terauchi M Hiramitsu S Akiyoshi M Associations among depression, anxiety and somatic symptoms in peri- and postmenopausal women. *J Obstet Gynaecol Res* 2013; 39:1007–1013. 23379427 10.1111/j.1447-0756.2012.02064.x

[21] Ye YB Tang XY Verbrugen MA Soy isoflavones attenuate bone loss in early postmenopausal Chinese women: a single-blind randomized, placebo-controlled trial. *Eur J Nutr* 2006; 45:327–334. 16763748 10.1007/s00394-006-0602-2

[22] Zung WW A self-rating depression scale. *Arch Gen Psychiatr* 1965; 12:63–70. 14221692 10.1001/archpsyc.1965.01720310065008

[23] Zung WW A rating instrument for anxiety disorders. *Psychosomatics* 1971; 12:371–379. 5172928 10.1016/S0033-3182(71)71479-0

[24] Loh FH Khin LW Saw SM The age of menopause and the menopause transition in a multiracial population: a nation-wide Singapore study. *Maturitas* 2005; 52:169–180. 16257608 10.1016/j.maturitas.2004.11.004

[25] Blümel JE Chedraui P Baron G Collaborative Group for Research of the Climacteric in Latin America (REDLINC). Menopausal symptoms appear before the menopause and persist 5 years beyond: a detailed analysis of a multinational study. *Climacteric* 2012; 15:542–551. 22530706 10.3109/13697137.2012.658462

[26] Agwu UM Umeora OU Ejikeme BN Patterns of menopausal symptoms and adaptive ability in a rural population in South-east Nigeria. *J Obstet Gynecol* 2008; 28:217–221. 18393024 10.1080/01443610801915637

[27] Gold EB Bromberger J Crawford S Factors associated with age at natural menopause in a multiethnic sample of midlife women. *Am J Epidemiol* 2001; 153:865–874. 11323317 10.1093/aje/153.9.865

[28] Oğurlu N Küçük M Aksu H Influence of employment status on menopausal symptoms. *Int J Gynaecol Obstet* 2011; 112:204–207. 21247563 10.1016/j.ijgo.2010.10.010

[29] Kapur P Sinha B Pereira BM Measuring climacteric symptoms and age at natural menopause in an Indian population using the Greene Climacteric Scale. *Menopause* 2009; 16:378–384. 19057415 10.1097/gme.0b013e31818a2be9

[30] Wang HL Booth-LaForce C Tang SM Depressive symptoms in Taiwanese women during the peri- and post-menopause years: Associations with demographic, health, and psychosocial characteristics. *Maturitas* 2013; 75:355–360. 23726260 10.1016/j.maturitas.2013.04.021

[31] Unsal A Tozun M Ayranci U Prevalence of depression among postmenopausal women and related characteristics. *Climacteric* 2011; 14:244–251. 20964551 10.3109/13697137.2010.510912

[32] Freeman EW Sammel MD Lin H Temporal associations of hot flashes and depression in the transition to menopause. *Menopause* 2009; 16:728–734. 19188849 10.1097/gme.0b013e3181967e16 PMC2860597

[33] World Health Organization. Ottawa charter for health promotion. 1986 http://www.euro.who.int/de/publications/policy-documents/ottawa-charter-for-health-promotion Accessed Dec 12, 2015.

[34] Leischik R Dworrak B Strauss M Plasticity of Health. *German J Med* 2016; 1:1–17.

[35] Mackenbach JP Stirbu I Roskam AJ Socioeconomic inequalities in health in 22 European countries. *N Engl J Med* 2008; 358:2468–2481. 18525043 10.1056/NEJMsa0707519

[36] Demakakos P Biddulph JP Bobak M Wealth and mortality at older ages: a prospective cohort study. *J Epidemiol Community Health* 2016; 70:346–353. 26511887 10.1136/jech-2015-206173 PMC4819652

[37] Wilkinson RG Unhealthy Societies: The Afflictions of Inequality. London: Routledge; 2002.

[38] Santosa A Schröders J Vaezghasemi M Ng N Inequality in disability-free life expectancies among older men and women in six countries with developing economies. *J Epidemiol Community Health* 2016; doi: 10.1136/jech-2015-206640 [Epub ahead of print]. 10.1136/jech-2015-206640 PMC5013163 26994068

[39] Cambois E Sole-Auro A Bronnum-Hansen H Educational differentials in disability vary across and within welfare regimes: a comparison of 26 European countries in 2009. *J Epidemiol Community Health* 2016; 70:331–338. 26546286 10.1136/jech-2015-205978

[40] Bucholz EM Ma S Normand SL Race, socioeconomic status, and life expectancy after acute myocardial infarction. *Circulation* 2015; 132:1338–1346. 26369354 10.1161/CIRCULATIONAHA.115.017009 PMC5097251

